# The effect of myeloablative radiation on urinary bladder mast cells

**DOI:** 10.1038/s41598-024-56655-5

**Published:** 2024-03-14

**Authors:** Jessica Smith, Jonathan Kah Huat Tan, Christie Short, Helen O’Neill, Christian Moro

**Affiliations:** 1 Faculty of Health Sciences and Medicine, Bond University, Gold Coast, QLD 4226 Australia; 2 Clem Jones Centre for Regenerative Medicine, Bond University, Gold Coast, QLD 4226 Australia

**Keywords:** Bladder, Radiotherapy

## Abstract

Radiation-induced cystitis is an inflammatory condition affecting the urinary bladder, which can develop as a side effect of abdominopelvic radiotherapy, specifically external-beam radiation therapy or myeloablative radiotherapy. A possible involvement of mast cells in the pathophysiology of radiation-induced cystitis has been indicated in cases of external-beam radiation therapy; however, there is no evidence that these findings apply to the myeloablative aetiology. As such, this study investigated potential changes to urinary bladder mast cell prevalence when exposed to myeloablative radiation. Lethally irradiated C57BL/6J mice that received donor rescue bone marrow cells exhibited an increased mast cell frequency amongst host leukocytes 1 week following irradiation. By 4 weeks, no significant difference in either frequency or cell density was observed. However mast cell diameter was smaller, and a significant increase in mast cell number in the adventitia was observed. This study highlights that mast cells constitute a significant portion of the remaining host leukocyte population following radiation exposure, with changes to mast cell distribution and decreased cell diameter four weeks following radiation-induced injury.

## Introduction

Radiation-induced cystitis is an inflammatory condition affecting the urinary bladder, which can develop as a side-effect of external beam radiation therapy (EBRT) on abdominopelvic cancers^1^ or myeloablative radiotherapy used in haematopoietic stem cell transplantation (HSCT) conditioning regimens^2,3^. Radiation directed at the urinary bladder is reported to induce inflammation of the urothelium^4–7^, damage to urinary bladder vasculature^5–8^, increased collagen deposition in the lamina propria^6,9–11^ and detrusor smooth muscle layers of the bladder^4–6,9^; though the mechanisms underpinning radiation-induced injuries are not yet known.

Current pre-clinical research has suggested a role for mast cells in the pathophysiology of radiation-induced cystitis^5,12–14^. In the healthy urinary bladder, mast cells are critical regulators of immunity^15–18^. They are distributed throughout all layers of the urinary bladder wall^18,19^ and are reported to be close to vasculature^20^. Histologically, mast cells are characterised by their metachromatic staining properties using the toluidine blue stain, though they can also be identified by flow cytometry through the expression of their characteristic markers: stem cell factor (CD117) and Fc receptor epsilon alpha (FcεRIα)^21–23^. Mast cell prevalence can be further contextualised in terms of the broader CD45^+^ leukocyte populations, such that they have been reported to constitute around 1% to 5% of all leukocytes in the urinary bladder^22^.

In the context of radiation-induced pathologies, mast cells have been associated with the development of fibrosis in the urinary bladder and in other tissues around the body^24–27^.

Through their degranulates, mast cells have been implicated in various physiological and pathological processes, including wound healing, angiogenesis, tissue repair and defence against pathogens^28^. In the urinary bladder, mast cells have been implicated in the pathogenesis of various disorders, such as urinary tract infections^29^, cancer^30^, contractile diseases^19,31^ and various forms of cystitis^19,32^. In such pathologies, abnormalities have been reported in the activation state, prevalence, and distribution of mast cells, suggesting their prominent role in many urinary bladder pathologies. Concerning radiation-induced cystitis, mast cells are believed to act by releasing cytokines such as transforming growth factor-β (TGF-β). TGF-β is of particular interest, as it can induce fibrosis by promoting fibroblast recruitment and proliferation^33^. Additionally, mast cells are believed to disrupt vasculature by increasing the permeability of blood vessels^34^ and playing a direct role in chemical cascades that result in radiation-induced injury^26^.

Previous literature has suggested a role for the urinary bladder mast cell in radiation-induced cystitis. However, these findings are only applicable to the EBRT aetiology and not the myeloablative aetiology^13,14,25,35,36^. The latter may differ from EBRT aetiology, in that a key characteristic of myeloablative radiotherapy is the destruction of proliferative cells in bone marrow. Specifically, myeloablation is known to disrupt haematopoiesis and subsequent recovery of leukocyte populations^37,38^ and may therefore disrupt the recovery of leukocytes in the urinary bladder following radiation exposure. Considering current research focusing on mast cells and their possible role in the pathophysiology of radiation-induced injuries to the urinary bladder, coupled with the uncertainty surrounding the impact of radiation exposure on leukocyte recovery, this study sought to clarify the effect of myeloablative radiation exposure on urinary bladder mast cells to draw conclusions on their possible involvement in disease.

## Results

### Leukocyte prevalence

**Figure 1:**
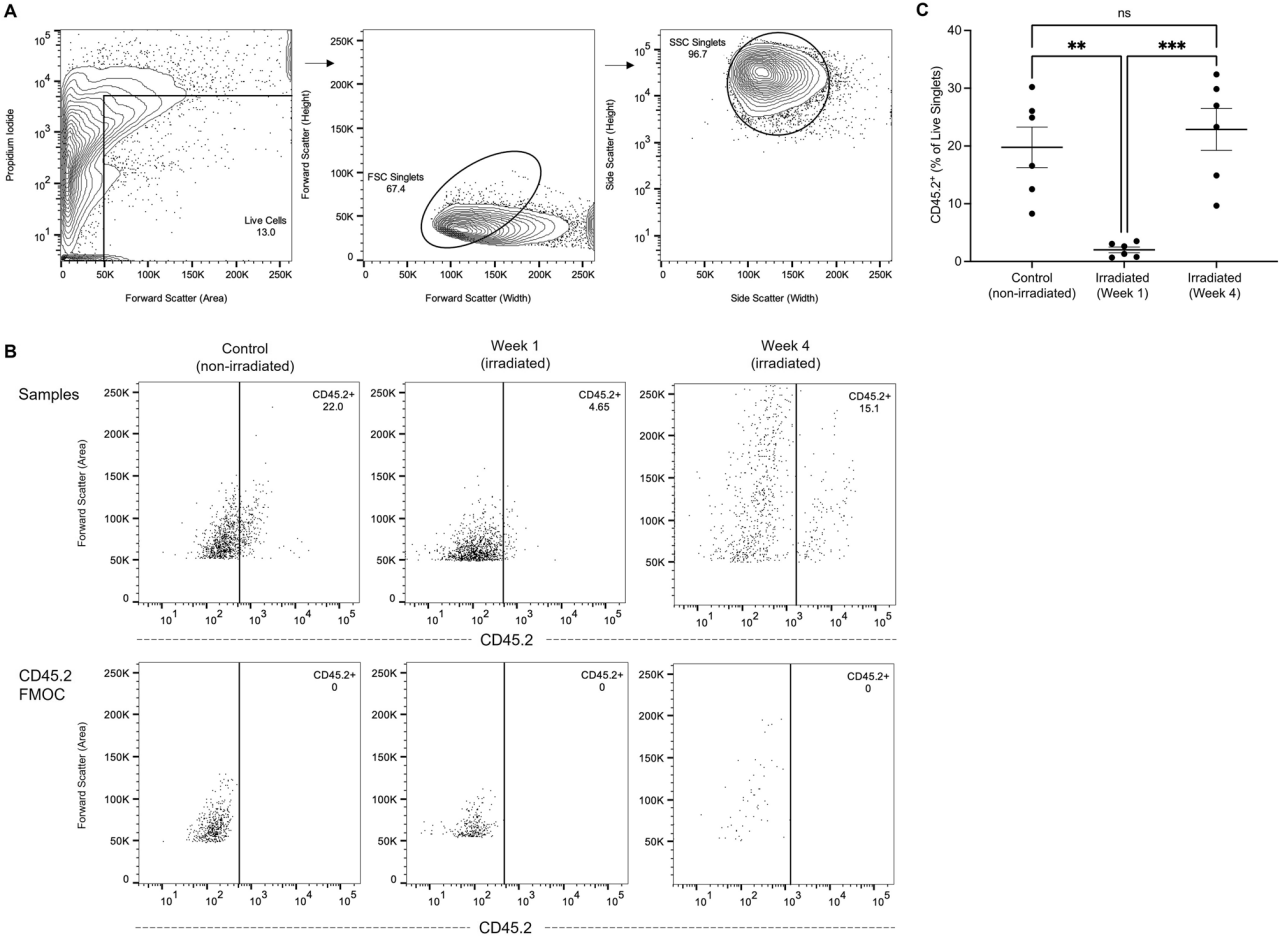
Representative gating of urinary bladder leukocytes. (**A**) Contour plots demonstrating the identification of live cells through the exclusion of propidium iodide-labelled cells, followed by forward scatter (FSC) and side scatter (SSC) doublet discrimination in a control urinary bladder sample. (**B**) Demonstrates the identification of host (CD45.2^+^) leukocytes in control (non-irradiated) and irradiated urinary bladders at one- and four-week time points, gated from live singlet populations, with CD45.2 FMOCs illustrated below. (**C**) Shows the frequency of host leukocytes in control and irradiated urinary bladders.

In non-irradiated urinary bladders, 19.76 ± 3.51% (mean ± SEM) of live singlet urinary bladder cells were CD45.2^+^. Following irradiation, host leukocytes decreased in frequency significantly after one week (2.00 ± 1.23%, *p* = 0.0018). Leukocyte prevalence did not significantly differ between control and irradiated bladders after four weeks (22.87 ± 3.63%, *p* = 0.7381), though it significantly differed between one- and four-week irradiation time points (*p* = 0.0004, Fig. 1C).

### Mast cell prevalence in the irradiated urinary bladder

**Figure 2:**
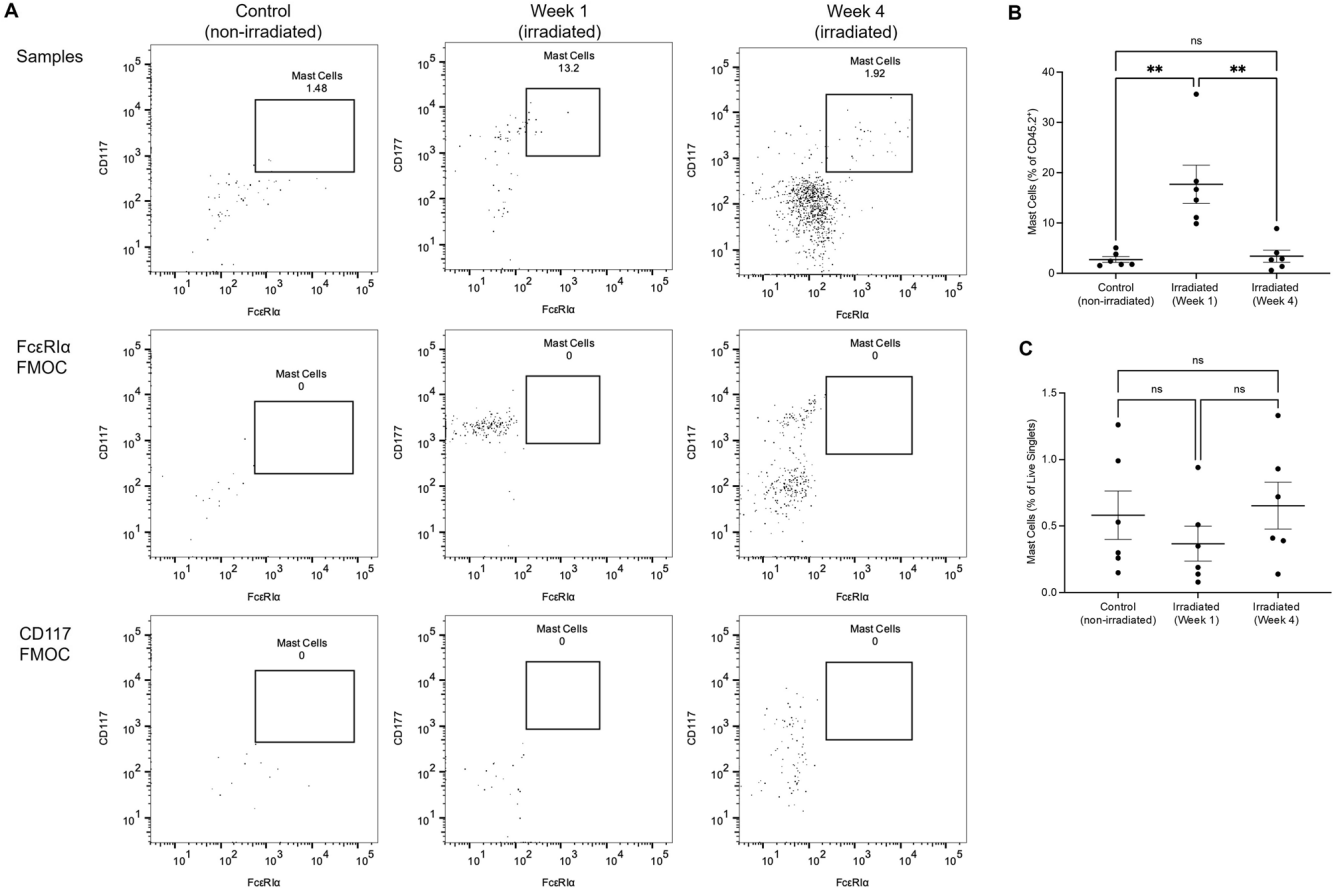
Identification of mast cells in the irradiated urinary bladder using flow cytometry. (**A**) Representative dot plots of mast cells from live, CD45.2^+^ singlet populations with FcεRIα and CD117 FMOCs are illustrated below. (**B**) Frequency of mast cells in host leukocytes in non-irradiated and irradiated urinary bladders amongst CD45.2^+^ cells. (**C**) Frequency of mast cells in live singlet populations.

Mast cells were identified by flow cytometry based on the gating strategy in Fig. 1. From the identification of CD45.2^+^ leukocytes, mast cells were identified based on their characteristic expression of CD117 and FcεRIα cell surface markers (Fig. 2). Mast cells were determined to constitute 2.73 ± 0.57% of live, CD45.2^+^ cells. One week following irradiation, mast cell frequency significantly increased to 17.69 ± 3.81% amongst CD45.2^+^ populations (*p* = 0.0011). Mast cell frequency was unchanged between non-irradiated and irradiated bladders at the fourth week (3.41 ± 1.2%, *p* = 0.9769) but decreased between one- and four-week post-irradiation time points (*p* = 0.0016) (Fig. 2B). Additionally, mast cell prevalence amongst all live cell singlet cells did not differ between non-irradiated and irradiated urinary bladder samples at one- (*p* = 0.6392) and four-week (*p* = 0.9494) time points (Fig. 2C).

**Figure 3:**
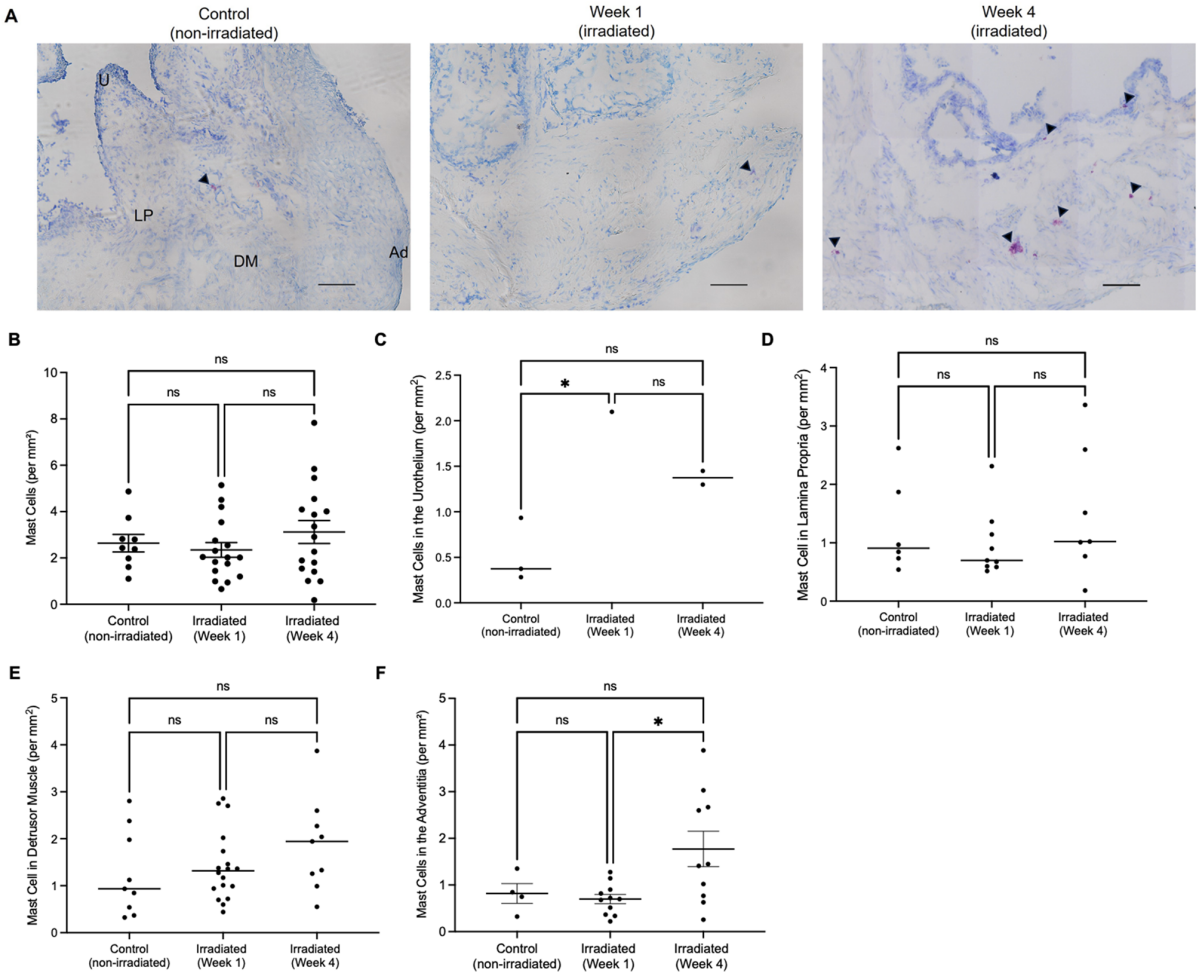
Toluidine blue staining of mast cells in the urinary bladder. (**A**) Representative images of non-irradiated and irradiated urinary bladders. Mast cells are indicated by black arrowheads. Scale bar represents 100 μm, × 20 magnification, *U* urothelium, *LP* lamina propria, *DM* detrusor muscle and *Ad* adventitia. (**B**) Number of mast cells per mm^2^ of urinary bladder tissue and number of mast cells per mm^2^ tissue in the (**C**) urothelium, (**D**) lamina propria, (**E**) detrusor muscle and (**F**) adventitia of the urinary bladder. Each dot represents mast cell numbers from one section of urinary bladder tissue.

Toluidine blue was also used to assess mast cell prevalence histologically (Fig. 3, revealing no statistically significant differences in the number of mast cells in non-irradiated and irradiated urinary bladders at one- (*p* = 0.9012) and four-week (*p* = 0.7500) time points (Fig. 3B).

Mast cell density per layer of the urinary bladder wall was also assessed between non-irradiated and irradiated urinary bladders (Fig. 3C–F). Mast cell density did not change in the lamina propria (3D) and detrusor muscle (3E) layers of the urinary bladder, however appeared to alter in the urothelium (Fig. 3C) and the adventitia (Fig. 3F). In the urothelium, mast cell density increased one week following radiation exposure compared to the non-irradiated controls (*p* = 0.0387), but not compared to the four-week timepoint (*p* = 0.1080) nor between one- and four-week timepoints (*p* = 0.2576). In the adventitia, mast cell density increased in the four-week timepoint compared to the one-week timepoint (*p* = 0.0167) but did not differ between control (*p* = 0.1043) or between control and one-week post-irradiation (*p* = 0.9658).

### Mast cell characteristics in the irradiated urinary bladder

**Figure 4:**
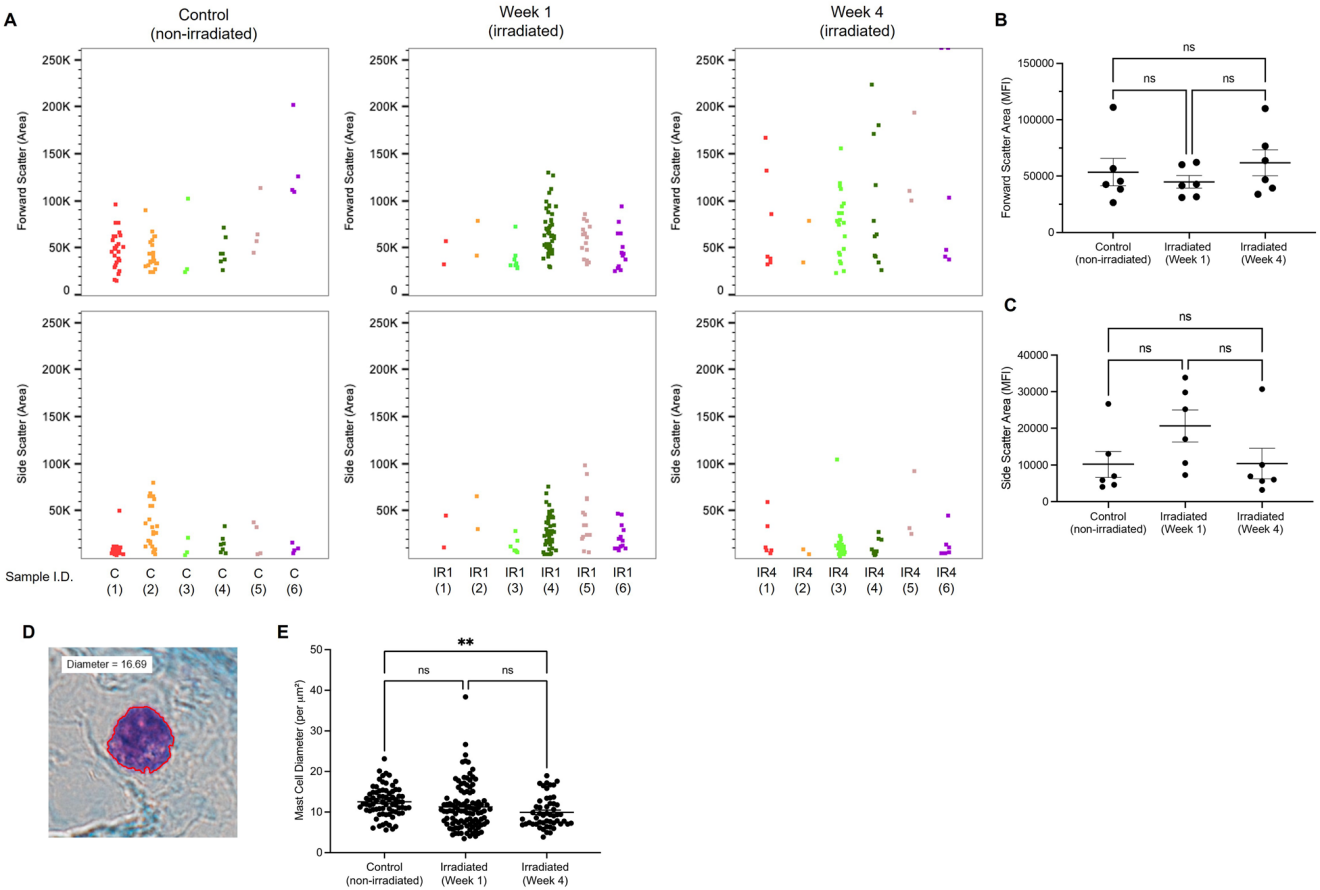
Irradiated mast cell morphology. (**A**) Concatenated flow cytometry data for forward scatter and side scatter parameters between non-irradiated and irradiated urinary bladders (n = 6 per group). (**B**) Median fluorescent intensity of forward scatter area and (**C**) median fluorescent intensity of side scatter area for each experimental group. (**D**) Representative image of mast cell stained with toluidine blue in the urinary bladder with annotated diameter measurement and (**E**) comparison of mast cell diameter between non-irradiated and irradiated bladders at one- and four-week time points. Each dot represents a single mast cell in (**A**) and (**E**), whereas (**B**) and (**C**) represent pooled data from non-irradiated and irradiated experimental samples.

Approximate size and internal complexity were measured using the forward scatter and side scatter parameters (via flow cytometry), and cell diameter (via microscopy) was also investigated in urinary bladder mast cell populations. Between non-irradiated and irradiated time points, there was no statistically significant difference in forward and side scatter median fluorescent intensities, despite apparent changes in forward scatter four weeks following radiation exposure (Fig. 4).

The equivalent diameter was also measured to further investigate potential alterations to mast cell characteristics. While there was no significant difference between non-irradiated and irradiated bladders one week following irradiation, a significant decrease in mast cell diameter was observed between non-irradiated and irradiated urinary bladders at four weeks following radiation injury (*p* = 0.0068, Fig. 4E).

## Discussion

Radiation-induced cystitis is an inflammatory condition affecting the urinary bladder and can develop as a side-effect of pelvic radiotherapy^1^ and in HSCT conditioning regimens, such as myeloablative radiotherapy^2,3^. Current research has identified a role for mast cells in the pathophysiology of this disease^14^. Although, current literature has focussed entirely on the EBRT aetiology of radiation-induced cystitis and not the myeloablative aetiology^14^, which was the focus of this investigation.

Firstly, the effect of myeloablative radiation on host leukocyte prevalence was investigated. One week following irradiation, host leukocytes decreased significantly in number (*p* = 0.0018) compared with non-irradiated controls (Fig. 1C), suggesting that myeloablative radiation has an immediate impact on leukocyte populations in the urinary bladder. This finding is consistent with the expected result of HSCT conditioning regimens, which aim to induce myeloablation and suppress the transplant recipient’s immune system to avoid allograft rejection^39^. As a result, HSCT conditioning regimens are known to disrupt haematopoiesis and subsequent recovery of leukocyte populations^37,38^. Additionally, this finding is supported by reports of decreasing numbers of CD4^+^ T-cells^40^ and CD3^+^ lymphocytes^13^ in the irradiated urinary bladder. Though, the full extent to which urinary bladder leukocytes are affected by radiation is not yet known, warranting further investigation.

Leukocyte depletion following myeloablative radiation exposure affects the bladder’s innate and adaptive immune systems and may also affect the bladder’s ability to recover from radiation-induced injuries. Of note, HSCT and its conditioning regimens are associated with adverse treatment outcomes, such as increased disease morbidity, prolonged hospitalisation^39,41^ and significant increases in non-relapse-related mortality^42^, though the exact cause underpinning these adverse outcomes is not clear. It is possible that leukocyte depletion may be related to such adverse treatment outcomes. To elaborate, the urinary bladder is continuously exposed to pathogens, such as uropathogenic *Escherichia coli*^43^ and the dormant BK virus^44^. Despite this, the healthy urinary bladder is generally resistant to infection. Following leukocyte depletion, the irradiated urinary bladder may not have adequate defences against such pathogens and thus may be susceptible to infection and subsequent disease. In fact, the reactivation of the BK virus following HSCT is recognised as a major pathological cause of cystitis following HSCT and its conditioning regimens^44,45^. It is likely that leukocyte depletion in the irradiated urinary bladder provides such pathogens with the opportunity to infect the HSCT recipient, subsequently contributing to adverse treatment outcomes, though more research is needed to establish a causative link.

Four weeks following radiation exposure, the frequency of host leukocytes returned to non-irradiated control levels (*p* = 0.7381), suggesting that the leukocyte populations in the bladder may recover from the effects of radiation over time (Fig. 1C). This finding is consistent with reports of leukocyte reconstitution occurring between 12 and 40 days post-HSCT, though in some instances leukocyte reconstitution can take months^46–48^. The initial decrease in leukocyte prevalence observed after one week may represent a transient response to the acute effects of irradiation, while the four-week time point may represent a more stable state of immune cell presence in the bladder, with a gradual recovery of leukocytes occurring between the one- and four-week time points.

Following this determination, the effect of myeloablative radiation on tissue-resident mast cells was then examined by flow cytometry and histology. Flow cytometric analysis demonstrated that mast cells constituted 3% of the healthy (non-irradiated) urinary bladder leukocyte populations (Fig. 2B), which was consistent with current literature^22^. After one week, the prevalence of mast cells within leukocyte populations increased significantly compared to non-irradiated bladders (*p* = 0.0011), although mast cell frequency did not differ between non-irradiated and four-week irradiated bladders (*p* = 0.9769) (Fig. 2B). This suggests that mast cells constitute approximately one-fifth of the entire immune cell population one week after radiation exposure, which may indicate their involvement in radiation-induced injuries in the urinary bladder. At four weeks post-irradiation, mast cell frequency was comparable to non-irradiated levels (*p* = 0.9769) in line with leukocyte reconstitution^46–48^. Histologically, mast cell numbers did not differ significantly when assessed per mm^2^ of tissue in both one- (*p* = 0.9012) and four-week (*p* = 0.7500) time points (Fig. 3B). Consistent with this evidence is that the percentage of mast cells amongst live singlet cells did not differ between non-irradiated and irradiated urinary bladder samples at one- (*p* = 0.6392) and four-week (*p* = 0.9494) time points (Fig. 2C). This finding is reinforced by previous evidence, which indicated that mast cell prevalence is unaffected by radiation in the urinary bladder^13,25^. Specifically, research conducted by Zwaans et al.^25^ and Podmolíková et al.^13^ found no statistically significant changes in the total number of mast cells in the irradiated bladder of Sprague–Dawley rats. With regard to the present study, these data suggest that while the percentage of mast cells increases in leukocyte populations one week following radiation exposure and returns to non-irradiated levels four weeks following radiation exposure, the percentage of mast cells does not change amongst the total number of live cells extracted from urinary bladder single cell suspensions. Additionally, these data illustrate that mast cells constitute a significant portion of the remaining leukocyte population in the urinary bladder and may therefore suggest a role in the recovery of the urinary bladder and potential pathologies following radiation injury.

Mast cell distribution remained unaffected in the lamina propria (Fig. 3D) and detrusor muscle (Fig. 3E) layers of the urinary bladder between non-irradiated, one- and four-week timepoints. These findings are partially supported by research conducted by Podmolíková et al.^13^. The aforementioned study indicated no statistically significant differences in mast cell numbers across the discrete layers of the urinary bladder wall—aligning with the lamina propria and detrusor muscle data presented in Fig. 3D and E, respectively. Additionally, a significant increase in mast cell density in the urothelium (Fig. 3C) and adventitia (Fig. 3F) was noted. In the urothelium, mast cell density increased one week following radiation exposure compared to the non-irradiated controls (*p* = 0.0387), but not compared to the four-week timepoint (*p* = 0.1080) nor between one- and four-week timepoints (*p* = 0.2576). This finding is contradicted by current literature, where a study conducted by Giglio et al.^49^ reported a significant decrease of mast cells in the urothelium (*p* < 0.05). The urothelium is known for its role in innate immunity^50^, and in the context of radiation-induced cystitis, histopathological reports indicate decreased urothelial cell numbers^4–7^, swelling^9^, irregular cell nucleation^5^, ulceration^5,9^ and compromised barrier function^51^ in the irradiated urinary bladder. An increase in mast cell density within the urothelium, indicated by the results of the present study, may suggest an involvement in such histopathological features noted in the irradiated bladder. Though this finding indicates statistical significance, scrutiny is warranted. To elaborate, while the density of mast cells per mm^2^ of tissue increased one week following radiation exposure, mast cells were detected in only one of six irradiated samples at this time point. In the remaining five samples, no mast cells were detected in the urothelium (Fig. 3C). Future studies may wish to reproduce the methods described in the present study with larger sample sizes to validate these findings.

In the adventitia, mast cell density increased between non-irradiated and irradiated bladders four weeks following irradiation (*p* = 0.0167) (Fig. 3F). Contextually, the adventitia of the urinary bladder connects the bladder to other organs of the abdominopelvic region; beyond acting as an interface between the bladder and other abdominopelvic organs, the adventitia’s functional significance is unknown. The urothelium, lamina propria and detrusor muscle layers, however, have physiological significance in the urinary bladder—from innate immunity^50^, signal transduction^52^ and micturition^53^, respectively. As a result, it is difficult to interpret this finding. It is possible that radiation may have an unbeknownst effect on the adventitia that would result in the aggregation of mast cells in this layer of the urinary bladder wall, though current literature in this area remains sparse. Ultimately, more research is needed to contextualise the significance of this finding.

Lastly, potential changes to mast cell diameter were investigated. While there was no significant difference in forward (Fig. 4B) and side scatter (Fig. 4C) parameters, a significant reduction in mast cell diameter was observed histologically between non-irradiated and irradiated urinary bladders four weeks (*p* = 0.0068) following radiation injury (Fig. 4E). Decreases in mast cell size may indicate a late response to radiation-induced injury or may indicate the infiltration of mast cell progenitors. With regard to the former, a study conducted by Burwen and Satir^54^ found that, upon stimulation, mast cell radius decreased by approximately 5%. Taken together with this finding, this may suggest that mast cells have a late response to radiation injury in the urinary bladder. Concerning the latter, a study conducted by Dahlin and Hallgren^55^ determined that mast cell progenitors, identified by their high expression of integrin β7, were smaller in diameter than mature mast cells. Thus, it is also possible that this decrease in diameter represents the infiltration of immature mast cells four weeks following irradiation. Of additional consideration is that these cells may be derived from transplanted donor cells, aligning with leukocyte reconstitution occurring between 12 and 40 days post-HSCT^46–48^.

Current pre-clinical research has demonstrated a potential role for mast cells in the pathophysiology of radiation-induced toxicities^5,12–14^; however, a clear link has not been established for the involvement of these immune cells in this disease. The present study is the first to demonstrate that mast cell prevalence does not change in response to myeloablative radiation exposure, and that mast cell frequency in broader leukocyte populations increases following acute radiation injury. These data suggest that mast cells represent a significant portion of the remaining leukocytes following acute radiation injury and may be involved in the early pathophysiology of radiation-induced cystitis. Future research should aim to investigate the effects of radiation on other urinary bladder leukocytes to better understand potential immunological mediators of radiation-induced cystitis. It is also recommended that future research aim to investigate potential causative links between adverse treatment outcomes, infection from opportunistic pathogens and leukocyte depletion to improve patient outcomes. Finally, future research should aim to investigate changes to mast cell distribution in urinary bladder disease, and to understand potential mechanisms underpinning morphological changes to mast cells following radiation exposure.

## Methods

### Animals and ethics approval

Female C57BL/6J and B6.SJL mice aged between 7 and 11 weeks were acquired from the Animal Resource Centre (Perth, Western Australia, Australia). Mice were housed and handled according to standard operating protocols approved by the University of Queensland (Brisbane, Australia) Animal Ethics Committee.

Euthanasia was performed by cervical dislocation. Animal ethics approval was granted by the University of Queensland Molecular Biosciences Animal Ethics Committee under a shared tissue agreement (BOND/ANRFA/162/20). All experiments were performed in accordance with relevant guidelines and regulations and are reported in accordance with ARRIVE Guidelines^56^.

### Animal irradiation and adoptive cell transfer

Recipient female C57BL/6J mice were exposed to 1.0 Gy/min of radiation by two opposing ^137^Cs γ-ray sources (Gammacell® 40 Extractor, Best Theratronics; Ottawa, Ontario, Canada). A split dose of 4.5 Gy and 5 Gy total body irradiation was given four hours apart, totalling 9.5 Gy, to induce myeloablation.

Within eight hours of irradiation, irradiated recipient mice received rescue bone marrow cells (1 × 10^5^) in combination with selected cell populations extracted from the femur and tibia of donor B6.SJL mice, described under *Magnetic Cell Depletion*. Rescue cells (1 × 10^5^) were resuspended in 300 µL of Dulbecco’s Modified Eagle Medium supplemented with 2% foetal calf serum (Sigma-Aldrich Corporation; St. Louis, Missouri, USA) and administered intravenously via the tail vein using a 26-gauge syringe. Irradiated mice received antibiotic water (neomycin sulphate, 1.1 g/L, and polymyxin B sulphate, 10^6^ U/L; Sigma-Aldrich Corporation) and were monitored daily using a score sheet. After one- and four-week time points, C57BL/6J mice were euthanised and urinary bladder tissue was harvested for assessment.

### Magnetic cell depletion

Multipotent progenitors and haematopoietic stem cells from donor B6.SJL mice were isolated to reconstitute the immune system and stem cell populations of irradiated C57BL/6J recipient mice. Bone marrow cell enrichment was achieved using the panel lineage-depletion antibodies as described in Table 1. Cells derived from bone marrow were prepared by ejecting the central marrow from the femur and tibia of both hind legs of naïve B6.SJL mice using 2 mL of ice-cold PBS with a 23G × ¼ syringe (Terumo Corporation; Shibuya, Tokyo, Japan) into a 14 mL conical centrifuge tube. Single-cell preparations were then centrifuged for five minutes (200G, 4 °C), and the supernatant was discarded. To remove erythrocytes, cell pellets were resuspended in 1 mL of red blood cell lysis buffer at a 1:10 dilution (eBioscience Incorporated; San Diego, California, USA) for five minutes at room temperature. Cells were then washed in 13 mL PBS and resuspended in 1 mL of staining buffer (phosphate buffered saline (PBS) containing 5% foetal bovine serum) (Sigma-Aldrich Corporation). Cells were then incubated for 10 min on ice, washed with 10 mL of staining buffer and resuspended in the remaining supernatant post-centrifugation. Magnetic beads (20 μL for 1 × 10^7^ cells; Miltenyi Biotech; Bergisch Gladbach, Germany) were added to the single-cell suspension, incubated for 10 min at 4 °C and centrifuged for five minutes (200G, 4 °C). The supernatant was discarded, and cells were resuspended in 2 mL of staining buffer in a culture tube (Becton, Dickinson and Company; Franklin Lakes, New Jersey, USA). Tubes were placed inside an EasyStep™ Magnet (STEMCELL Technologies; Vancouver, British Columbia, Canada) for 10 min. Flow-through cell fractions were decanted into a second culture tube and placed into the magnet for the second round of cell depletion. The negative cell fraction was decanted into a 14 mL conical centrifuge tube (TPP Techno Plastic Products AG; Transadingen, Switzerland), resuspended in 13 mL staining buffer, and then centrifuged for 5 min (200G, 4 °C). Supernatant was discarded, and cells were resuspended in 1 mL of staining buffer.

**Table 1:** Lineage depleting antibody panel for bone marrow.

Antibody (conjugation)	Clone	Isotype	Concentration	Supplier
CD16/13-purified (Fc block)	93	Rat IgG_2a_, λ	0.5 mg/mL	Biolegend
Ly6G-ly6C (Gr-1) (biotin)	RB6-8C5	Rat IgG_2b_, κ	0.5 mg/mL	Biolegend
CD11c (biotin)	N418	Armenian Hamster IgG_1_, κ	0.5 mg/mL	Biolegend
NK1.1 (biotin)	PK136	Rat IgG_2a_, κ	0.5 mg/mL	Biolegend
CD48 (biotin)	HM48-1	Armenian Hamster IgG_1_, κ	0.5 mg/mL	eBioscience
CD3ε (biotin)	145-2C11	Armenian Hamster IgG_1_, κ	0.5 mg/mL	BD Pharmingen
CD19 (biotin)	6D5	Rat IgG_2a_, κ	0.5 mg/mL	Biolegend

### Flow cytometry

Bladder tissue was harvested and prepared from C57BL6/J female mice to isolate tissue-resident immune cells. Tissue was cut into small pieces and enzymatically digested in a 14 mL conical centrifuge tube containing 400 μL of Collagenase IV (10 µg/mL; Sigma-Aldrich Corporation) and 1500 μL of Dulbecco’s Modified Eagle Medium (DMEM; Sigma-Aldrich Corporation). The tube was incubated at 37 °C for 10 min with constant rotation. 400 μL of Collagenase D (10 µg/mL; Sigma-Aldrich Corporation), 80 μL of deoxyribonuclease I from bovine pancreas. Type II-S (DNAse; 10 µg/mL; Sigma-Aldrich Corporation) and 11.62 mL of DMEM was added to the same tube and incubated for a further 20 min. Digested tissue was filtered through a 100 μm cell strainer (Greiner Bio-One; Kremsmünster, Upper Austria, Austria) into a new 14 mL conical centrifuge tube and centrifuged for 5 min (200G, 4 °C). The supernatant was then discarded. The cell pellet was resuspended in 1 mL of PBS supplemented with 2% bovine serum albumin (Sigma-Aldrich Corporation).

Cells were aliquoted into a 96-well U-bottom plate (TPP Techno Plastic Products AG; Trasadingen, Switzerland) and centrifuged for five minutes at 4 °C for 200G, after which supernatant was discarded. Wells were labelled with 10 μL antibody solution, prepared by diluting each antibody 1:100 in staining buffer. 10 μL of antibody solution was added to each well and then incubated for 10 min on ice with protection from light. Stained cells were then washed with 150 μL of staining buffer. Samples were then resuspended in 150 μL of staining buffer and transferred into 5 mL Falcon round-bottom tubes (Becton, Dickinson and Company; Franklin Lakes, New Jersey, USA) for flow cytometric analysis.

CD45 is a pan-leukocyte marker that detects almost all haematopoietic-derived cells, with the exception of mature erythrocytes^57^. The CD45.2 isotype of the pan-leukocyte marker was used to identify haematopoietic cells from C57BL/6J (host) mice^58,59^. Mast cells were also identified based on the expression of CD45, CD117 and FcεRIα outlined in Table 2. Propidium iodide (Sigma-Aldrich Corporation) was used to discriminate live and dead cells in all populations. Flow cytometric analysis and cell sorting were performed on a BD FACSAria™ Fusion (Becton Dickinson; Franklin Lakes, New Jersey, USA). Single-colour controls were used to adjust compensation for spectral overlap, and cell population gating was based on a series of fluorescence minus-one control antibody mixtures. Flow cytometric data was analysed using FlowJo (v10.8.1; FlowJo LLC; Ashland, Oregon, USA).

**Table 2:** Antibodies for mast cell detection.

Antibody	Fluorochrome	Clone	Isotype	Concentration	Supplier
FcεRIα	PE	MAR-1	Armenian Hamster IgG	0.2 mg/mL	BioLegend
CD117	aF647	2B8	Rat IgG2b, κ	0.5 mg/mL	BioLegend
CD45.2	PB	104	Mouse (SJL), IgG2a, κ	0.5 mg/mL	BioLegend

*aF647* Alexa Fluorochrome 647 dye, *FITC* fluorescein isothiocyanate, *PB* pacific blue, *PE* phycoerythrin. Antibodies were diluted at 1:100 in staining buffer.

### Toluidine blue staining

Toluidine blue was used to determine mast cell distribution and size in the non-irradiated and irradiated urinary bladder. Mast cells were identified by light microscopy based on their metachromatic appearance and violet colouration. To achieve this, tissue was harvested and prepared from C57BL6/J female mice. Tissue extracted from C57BL/6J mice was frozen in Tissue-Tek O.C.T. compound (Sakura Finetek USA Inc; Torrance, California, U.S.A). Three serial sections (10 μm thickness) were sectioned per urinary bladder using a Leica CM1800 Cryostat (Leica Microsystems GmbH; Wetzlar, Germany) and placed on Starfrost Advanced Adhesive slides (ProSciTech; Kirwan, Queensland, Australia).

Urinary bladder sections were first rehydrated through sequential five-minute immersions of 100%, 95% and 70% ethanol (w/v) (Sigma-Aldrich). Sections were then submersed for 2-min in distilled water and stained with toluidine blue (pH 2.2) for a further 2-min. Slides were washed with distilled water and then quickly dehydrated in 95% (twice) and 100% (twice) ethanol. After mounting, tissues were imaged using a live cell microscope with a brightfield camera (Nikon Eclipse Ti2-E; Nikon Corporation; Tokyo, Japan).

### Statistical analysis

Animal numbers for experimental studies were based on power calculations using pilot data of control urinary bladders and were reviewed in consultation with a biostatistician. Mast cell density was reported out of all three sections per sample by mm^2^ of bladder tissue. The equivalent diameter was calculated by NIS-Elements Advanced Research software (v4.0, Nikon Corporation; Tokyo, Japan) and reported as mast cell diameter in μm. To compare non-irradiated and irradiated controls, an ordinary one-way ANOVA with Tukey’s multiple comparisons test was used. Statistics were performed using Prism 9 for macOS (v9.3.0, GraphPad Software; San Diego, California, USA).

## Data Availability

Data used in this study will be made available from the corresponding author upon reasonable request.
